# Use of rehabilitation services by persons with disabilities in Brazil: A multivariate analysis from Andersen’s behavioral model

**DOI:** 10.1371/journal.pone.0250615

**Published:** 2021-04-29

**Authors:** Arthur de Almeida Medeiros, Maria Helena Rodrigues Galvão, Isabelle Ribeiro Barbosa, Angelo Giuseppe Roncalli da Costa Oliveira

**Affiliations:** 1 Integrated Health Institute, Federal University of Mato Grosso do Sul, Campo Grande, Mato Grosso do Sul, Brazil; 2 Graduate Program in Public Health, Federal University of Rio Grande do Norte, Natal, Rio Grande do Norte, Brazil; Università degli Studi di Perugia, ITALY

## Abstract

**Background:**

For many years, discussions about health care for people with disabilities (PwD) in Brazil have not been treated as a priority; however, based on the advances made at the beginning of this century, new policies have been developed with the aim of improving access of these people to health services. Therefore, the aim of this study was to analyze how individual characteristics and contextual indicators are associated with access to rehabilitation services for PwD in Brazil.

**Methods:**

A multivariate analysis was performed based on data from the National Health Survey 2013, considering access to rehabilitation services by PwD as the primary outcome and individual and contextual factors selected from Andersen’s behavioral model as independent variables. The contextual variables were reduced to two composite indicators (1-primary health care coverage and unfavorable socioeconomic conditions, and 2-economic inequality) from the analysis of the principal components. Poisson regression analysis with robust variance was performed to estimate the prevalence ratio (PR) and the respective 95% confidence interval (95%CI).

**Results:**

Access to rehabilitation services by PwD was more prevalent in people aged 0 to 17 years (PR = 3.28; 95%CI 2.85–3.78), who are illiterate (PR = 1.24; 95%CI 1.09–1.40), whose socioeconomic level is A or B (PR = 1.60; 95%CI 1.35–1.88), who have health insurance (PR = 1.31; 95%CI 1.15–1.49), who have severe limitations (PR = 3.09; 95%CI 2.64–3.62), who live in states with a good offer of Specialized Rehabilitation Centers, both type II (PR = 1.20; CI95% 1.08; 1.33) and type IV (PR = 1.29; CI95% 1.15; 1.44), and who have greater coverage of primary health care, but unfavorable socioeconomic conditions (PR = 1.15; CI95% 1.03–1.28).

**Conclusion:**

The results clarify the social inequities that exist regarding access to rehabilitation services for PwD in Brazil and highlight the need to formulate and implement public policies that guarantee the realization of the rights of these people.

## Introduction

The World Health Organization (WHO) [1] recognizes that disability is complex, dynamic, and multidimensional and that it is an evolving concept that cannot be treated in a dichotomized way, although debates about this concept have, for many years, occurred in the sense of building a definition based on the medical or social model [2]. In this sense, the incorporation of the International Classification of Functionality, Disability, and Health in the context of this discussion reinforces that disability is due to the interaction between health problems and contextual factors, and WHO therefore states that “disability results from interaction between people with disabilities and behavioral and environmental barriers that prevent their full and effective participation in society on an equal basis” [1].

In Brazil, the Brazilian Law for the Inclusion of Persons with Disabilities, also known as the Statute for Persons with Disabilities, considers persons with disabilities (PWD) “to have a long-term physical, mental, intellectual or sensory impairment, which, in interaction with one or more barriers, can obstruct their full and effective participation in society on equal terms with other people” [3]. It is a legal framework that, in addition to expanding the view on PWD, seeks to promote inclusion with guaranteed fundamental rights, accessibility, and access to justice.

The World Report on Disability estimates that approximately 15% of the population has a disability, which is equivalent to more than 1 billion people [1]. In Brazil, data from the 2010 Census regarding PWD were reviewed in 2018 based on the considerations made by the Washington Group for Statistics on People with Disabilities. It is a group established under the United Nations Statistics Commission whose premise is to standardize population-based research definitions, concepts, and methodologies so that the results obtained are reliable for comparing statistics between different countries, and the new results indicate that the prevalence of PWD in the country is 6.7% [4].

In view of the expressive data related to the number of PWD in Brazil, the debate about the organization of health services and the way in which this population has access to health services has become essential. The planning of care actions for PWD in the country for many years has fallen short of its due importance, and proof of this was the initiative proposed by the Ministry of Health of Brazil that dealt with the issues related to this theme in a punctual and fragmented manner [5].

However, from the social and political advances that took place at the beginning of the 21st century related to PWD, both at the international and national levels, increased discussions related to this theme were observed by the Brazilian government, which in 2011 resulted in the launch of Living without limits: National Plan for the Rights of Persons with Disabilities, a program that involves inter-ministerial coordination whose actions are based on the axes of health care, access to education, social inclusion, and accessibility [5,6].

From the launch of the Living without limits plan and considering Ordinance No. 4.279/2010, which established guidelines for the organization of the Health Care Network within the scope of the Unified Health System (SUS), the Ministry of Health of Brazil created the Health Network Health Care for Persons with Disabilities (RCPCD), which provides for the organization of services considering primary health care, specialized care, hospital care, and urgent and emergency care, and whose health care points are articulated in such a way that establishes the processes and flows necessary for the effectiveness of comprehensive, universal, and equitable care for this population [5–8].

As of 2012, the health care of PWD took on a new perspective, especially in the actions developed in specialized care, with a view to create Specialized Rehabilitation Centers (CER). These establishments assumed a central role in the organization of the RCPCD because they constitute a point of integration of the actions developed in the network and because they offer care to two, three, or four types of rehabilitation (physical, hearing, visual, and intellectual), thus breaking with the fragmented care logic and restriction to a specific type of disability [5].

Although the RCPCD represents an advance for the care of PWD, it is necessary to develop research that seeks to understand the impact of the organization of this network in the care offered to this population, especially with regard to the access of these people to health equipment, bearing in mind that the concept of access is multifaceted, consisting of economic and non-economic factors, and has been defended based on the principles of social justice and equity and, therefore, must be analyzed considering the moment of analysis, since as societies evolve, new needs are identified [9].

For the development of this research, Andersen’s behavioral model was adopted as a theoretical framework to assess access, defined as the use of health services itself, and analyze the individual and contextual factors that facilitate or prevent use. [10,11].

A recent literature review that sought to analyze access to health services by PWD in low- and middle-income countries identified that the use of health services is greater among PWD when compared to the others and that the main barriers to access are related to finances, transport, and relationship with the health team [12].

The literature highlights the importance and the need to conduct population-based research to establish a national health information system that, in addition to providing knowledge about the profile of the population and their health situation, enables health system evaluation and monitoring [13]. In this sense, the National Health Survey (PNS), carried out between August 2013 and February 2014, presents itself as an important tool for the knowledge and assessment of the health situation of PWD. In view of this, the objective of this study was to analyze how individual characteristics and contextual indicators are associated with access to rehabilitation services for people with disabilities in Brazil.

## Method

### Participants and database

This study was conducted based on data obtained by the National Health Survey 2013 (PNS), which is a household-based survey carried out in the national territory that sought to characterize the lifestyle and health situation of Brazilians, constituting it as part of the Integrated System of Home Research (SIPD) of the Brazilian Institute of Geography and Statistics (IBGE) [14].

PNS uses the SIPD Master Sample, as it allows greater territorial coverage. Sampling was carried out by clusters in three stages using a simple random sample. The first stage was characterized by the selection of primary sampling units (UPA), composed of census tracts. The second stage was the selection of the number of private households within each UPA, which ranged from 10 to 14; and the third stage was the selection of a resident aged 18 or over per household [15]. Ultimately, 81,254 households were selected, of which 69,994 were occupied. A total of 64,438 home interviews and 60,202 interviews were conducted with residents aged 18 or over [16].

For this research, the study population was defined as people who reported the presence of at least one disability and, therefore, respondents who answered affirmatively to one or more of the following questions were considered: G01 (do you have intellectual disability?), G06 (do you have a physical disability?), G014 (do you have hearing impairment?), and G021 (do you have visual impairment?).

Thus, the population of this study comprised 13,659 individuals, which corresponds to the number of residents who have at least one type of disability.

### Characterization of variables

#### Dependent variable

The dependent variable or primary outcome of the study, called “access to rehabilitation services,” was composed of individuals with at least one disability and the experience of attending at least one rehabilitation service.

The dependent variable was constructed considering the affirmative answers to at least one of the following questions: G05 (do you attend any rehabilitation service due to intellectual disability?), G010 (do you attend any rehabilitation service due to physical disability?), G018 (do you attend any rehabilitation service due to hearing impairment?), and G027 (do you attend any rehabilitation service due to visual impairment?). The dependent variable was dichotomized into: PwD who had no access to any rehabilitation service, and PwD who had access to at least one rehabilitation service.

#### Independent variables

**Fig 1:**
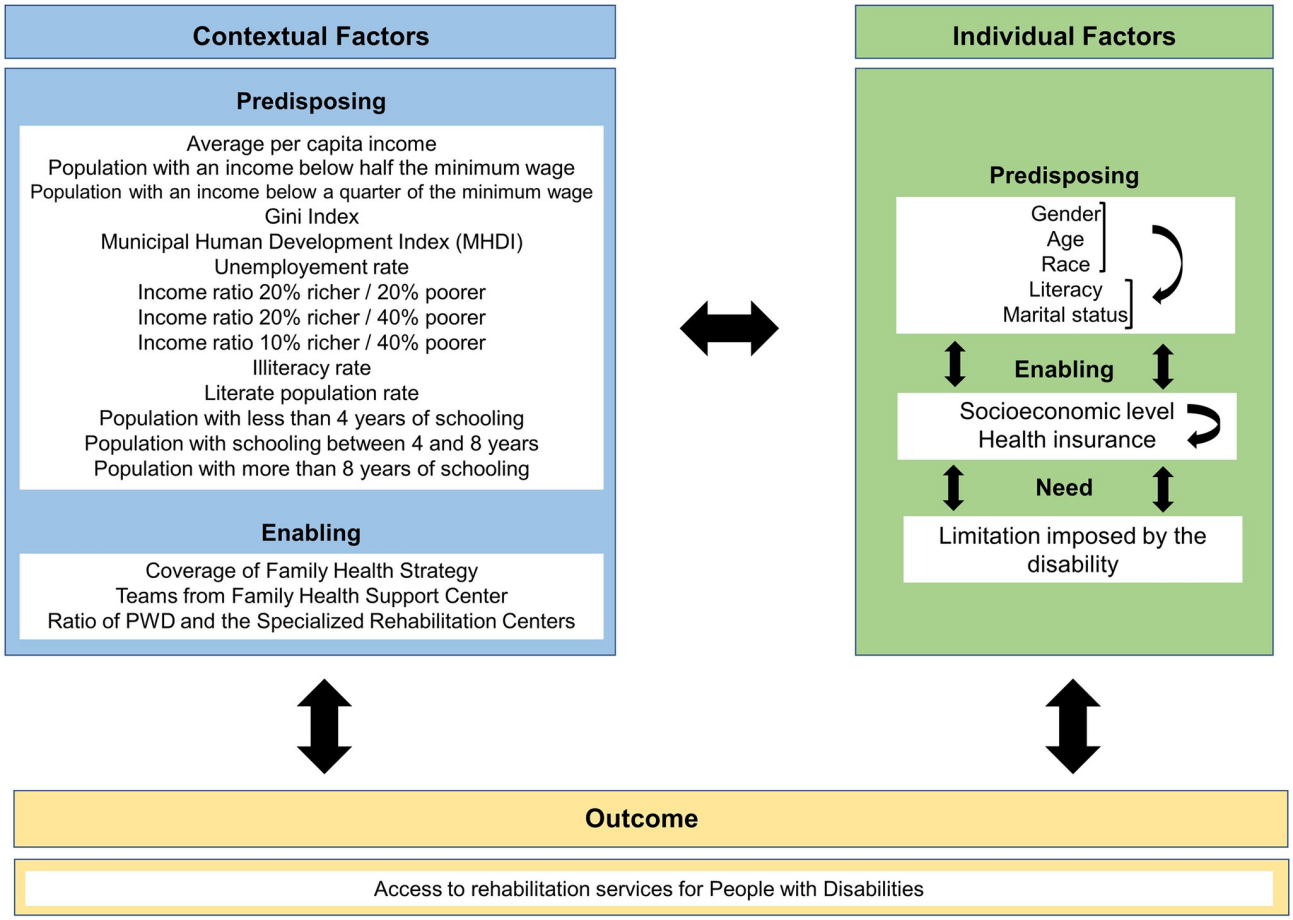
Access concept framework. Source: Adapted from Andersen [10].

For the selection of independent variables, Andersen’s behavioral model was adopted (Fig 1).

For individual predisposing factors, gender (male or female), age (stratified into age groups), race (white or non-white), literacy (yes or no), and marital status (single, married, separated, or widowed) were considered. The individual facilitating factors selected were having health insurance (yes or no) and the socioeconomic level classified according to the Brazil Criteria for Economic Classification (A, B, C, D, or E) [17]. The limitation imposed by the disability (without limitation, mild limitation, or severe limitation) was considered as an individual factor of need.

The answers to questions G004, G009, G017 and G026 were considered for the construction of the variable related to the limitation imposed by disability, which address, respectively, how intellectual disability, physical disability, hearing impairment and visual impairment impact on achievement usual activities. In the case of people with multiple disabilities, the highest level of limitation imposed by one of the reported disabilities was considered.

Regarding the contextual variables, the average per capita income, the proportion of the population with an income below half the minimum wage, the proportion of the population with an income below a quarter of the minimum wage, the Gini index, the Municipal Human Development Index (MHDI), the unemployment rate, the income ratio between the richest 20% and the poorest 20%, the income ratio between the richest 20% and the poorest 40%, the income ratio between the richest 10% and the poorest 40%, the illiteracy rate, the literate population rate, the proportion of the population with less than 4 years of schooling, the proportion of the population with schooling between 4 and 8 years, and the proportion of the population with more than 8 years of schooling were selected as predisposing factors. The contextual facilitating factors listed were the rate of coverage of the Family Health Strategy (FHS), the rate of the Family Health Support Center teams (Modality I, II, and II) per 1,000 inhabitants, and the ratio between the number of people with disabilities and the number of Specialized Rehabilitation Centers (CER), calculated according to type (II, III, and IV).

The individual independent variables were collected from the PNS questionnaire itself, and the contextual variables were obtained from the websites of the National Register of Health Establishments (CNES), the Institute of Applied Economic Research (IPEA), and the Department of Informatics of the Unified Health System (DATASUS) and the Primary Care Information and Management System (E-Gestor) (Table 1).

**Table 1:** Description of the individual and contextual variables of the study and adaptation strategies for the analysis model. Brazil, 2013.

Variable	Source Reference Year	Description	Original Categorization (Adapted Categorization)
Gender	National Health Survey (PNS) 2013	Gender	Male Female
Race	National Health Survey (PNS) 2013	Self-reported skin color	White (White) Black (Non-White) Asian (Non-White) Brown (Non-White) Indian (Non-White)
Age	National Health Survey (PNS) 2013	Age, in years, at the time of the interview	Age in years categorized into age groups. 0 to 17 years 18 to 29 years 30 to 39 years 40 to 59 years 60 years or older
Literacy	National Health Survey (PNS) 2013	Answer to the question "Can you read and write?"	Yes No
Socioeconomic Level	National Health Survey (PNS) 2013	The “Brazil Criteria for Economic Classification” is based on the possession of goods, such as a car, computer, refrigerator, TV, washing machine, DVD player, microwave, motorcycle, and cell phone. There is also a bathroom, a maid, and the educational level of the head of the family. Each item has different weights, and the variable represents the family’s purchasing power.	The score is defined between 0 and 100 points and divided into classes as follows: A– 45 to 100 (A and B) B1–38 to 44 (A and B) B2–29 to 37 (A and B) C1–23 to 28 (C) C2–17 to 22 (C) D and E– 0 to 16 (D and E)
Marital Status	National Health Survey (PNS) 2013	Marital status	Married Separated Widowed Single
Health Insurance	National Health Survey (PNS) 2013	Answer to the question "Do you have any health plan (medical or dental), private, company or public agency?"	Yes No
Limitation	National Health Survey (PNS) 2013	Degree in which the disability limits the habitual activities (such as going to school, playing, working, etc.) of the resident.	Does not limit (Without limitation) A little (Mild limitation) Moderately (Mild limitation) Intensely (Severe limitation) Very intensely (Severe limitation)
Ratio between the number of people with disabilities and the number of Specialized Rehabilitation Centers (CER) Type II	National Register of Health Facilities (CNES) 2014	Proportion of people with disabilities (PWD), according to the 2010 population census, in relation to the number of specialized rehabilitation centers type II.	Numerical value categorized from the median in: Up to 637,105 PWD/CER II (Low) More than 637,106 PWD/CER II (High)
Ratio between the number of people with disabilities and the number of Specialized Rehabilitation Centers (CER) Type III	National Register of Health Facilities (CNES) 2014	Proportion of people with disabilities (PWD), according to the 2010 population census, in relation to the number of specialized rehabilitation centers type III.	Numerical value categorized from the median in: Up to 882,022 PWD/CER III (Low) More than 882,023 PWD/CER III (High)
Ratio between the number of people with disabilities and the number of Specialized Rehabilitation Centers (CER) Type IV	National Register of Health Facilities (CNES) 2014	Proportion of people with disabilities (PWD), according to the 2010 population census, in relation to the number of specialized rehabilitation centers type IV.	Numerical value categorized from the median in: Up to 2,336,027 PWD/CER IV (Low) More than 2,336,028 PWD/CER IV (High)
Average Per Capita Income	Applied Economic Research Institute (IPEA) 2010	Ratio between the sum of the income of all individuals living in permanent private households and the total number of these individuals.	*Continuous variable
Gini Index	Applied Economic Research Institute (IPEA) 2010	It measures the degree of inequality in the distribution of individuals according to household income per capita. Its value varies from 0, when there is no inequality (the per capita household income of all individuals has the same value), to 1, when inequality is maximum (only one individual holds all income). The universe of individuals is limited those living in permanent private households.	*Continuous variable
Municipal Human Development Index (MHDI)	Applied Economic Research Institute (IPEA) 2010	Municipal Human Development Index refers to the geometric mean of the indices of the dimensions Income, Education, and Longevity, with equal weights.	*Continuous variable
Unemployment rate	Department of Informatics of the Unified Health System (DATASUS) 2010	Proportion of the economically active resident population aged 16 and over who is unemployed in the reference week, in a given geographical space, in the year considered.	*Continuous variable
Population with income below half the minimum wage	Department of Informatics of the Unified Health System (DATASUS) 2010	Proportion of resident population with per capita monthly household income of up to half the minimum wage, in a given geographic space, in the year considered	*Continuous variable
Population with income below a quarter of the minimum wage	Department of Informatics of the Unified Health System (DATASUS) 2010	Proportion of resident population with per capita monthly household income of up to a quarter of the minimum wage, in a given geographical space, in the year considered.	*Continuous variable
Income Ratio 20% richer/20% poorer	Department of Informatics of the Unified Health System (DATASUS) 2010	Number of times that the aggregate income of the upper fifth of the income distribution (20% richer) is greater than the income of the lower fifth (20% poorest) in the population residing in a given geographical space, in the year considered. Per capita household income was considered the sum of the monthly income of the residents of the household, in reais, divided by the number of its residents.	*Continuous variable
Income Ratio 20% richer/40% poorer	Applied Economic Research Institute (IPEA) 2010	Measure of the degree of inequality that exists in the distribution of individuals according to per capita household income. It compares the average per capita income of individuals belonging to the richest fifth in this distribution with the average per capita income of individuals belonging to the two poorest fifths. The universe of individuals is limited to those who live in permanent private households.	*Continuous variable
Income Ratio 10% richer/40% poorer	Applied Economic Research Institute (IPEA) 2010	Measure of the degree of inequality that exists in the distribution of individuals according to per capita household income. It compares the average per capita income of individuals belonging to the richest tenth of that distribution with the average per capita income of individuals belonging to the poorest four tenths. The universe of individuals is limited to those who live in permanent private households.	*Continuous variable
Illiteracy	Department of Informatics of the Unified Health System (DATASUS) 2010	Percentage of people aged 15 or over who do not know how to read and write at least one simple note, in the language they know, in the total resident population of the same age group, in a given geographical space, in the year considered.	*Continuous variable
The literate population rate	Department of Informatics of the Unified Health System (DATASUS) 2010	Percentage of people aged 15 or over who can read and write at least one simple note, in the language they know, in the total resident population of the same age group, in a given geographical space, in the year considered.	*Continuous variable
The proportion of the population with less than 4 years of schooling	Department of Informatics of the Unified Health System (DATASUS) 2010	Percentage of people aged 15 or over who have less than 4 years of schooling.	*Continuous variable
The proportion of the population with schooling between 4 and 8 years	Department of Informatics of the Unified Health System (DATASUS) 2010	Percentage of people aged 15 or over who have between 4 and 8 years of schooling.	*Continuous variable
The proportion of population with more than 8 years of schooling	Department of Informatics of the Unified Health System (DATASUS) 2010	Percentage of people aged 15 and over who have more than 8 years of schooling.	*Continuous variable
Coverage of the Family Health Strategy	Primary Care Management and Information System (E-Gestor) 2014	The calculation of the covered population is made from the population base published in ministerial decree and used to determine the federal government’s financial transfer to the municipality.	*Continuous variable
Teams from the Family Health Support Center—Modality I (NASF-AB I)	Primary Care Management and Information System (E-Gestor) 2014	Number of NASF-AB teams—modality I divided by the total population of the state according to the 2010 census for every 100 thousand inhabitants.	*Continuous variable
Teams from the Family Health Support Center—Modality II (NASF-AB II)	Primary Care Management and Information System (E-Gestor) 2014	Number of NASF-AB teams—modality II divided by the total population of the state according to the 2010 census for every 100 thousand inhabitants.	*Continuous variable
Teams from the Family Health Support Center—Modality III (NASF-AB III)	Primary Care Management and Information System (E-Gestor) 2014	Number of NASF-AB teams—modality III divided by the total population of the state according to the 2010 census for every 100 thousand inhabitants.	*Continuous variable

### Statistical analysis

The PNS database and the contextual independent variables were aggregated using the deterministic linkage technique with reference to the units of the federation.

For all variables, the presence of missing data and outliers was analyzed, and it was verified that the variables, marital status and literacy presented, respectively, 3.86% and 1.16% of the missing data. According to Hair et al. [18], missing data below 10% can be ignored. For the other variables, the presence of missing data and outliers was not identified.

In view of the list of contextual independent variables, it was decided to reduce them based on the factor analysis of principal components. For the construction of the composite indicator, a correlation analysis was performed between the independent variables, of which 14 variables had a correlation coefficient between ± 0.3 and ± 0.8, with p≤0.05, and met the criteria for inclusion in the model for factor analysis.

The model proved to be suitable for performing factor analysis with a Kaiser–Meyer–Olkin index (KMO) value equal to 0.803 and Bartlett’s sphericity test with significance *p*<0.001, thus rejecting the hypothesis that the correlation matrix is an identity matrix. The variables that presented a sample adequacy measure (MSA) and commonality greater than 0.5 were maintained in the model. Factors with a latent root greater than 1 and total explained variance greater than 70% were extracted, and the model adopted was the principal component analysis. To obtain the rotating component matrix, the Varimax orthogonal rotation was chosen [19].

Finally, two factors were extracted whose sum of the total explained variance was 84.344%. The rotational factor loads, the sample adequacy measure (MAS), and the commonality of each variable, according to the factors generated, are described in Table 2.

**Table 2:** Final model of the factor analysis with description of the factor loads, measure of sample adequacy, and commonality of the included variables, and latent root, percentage of explained and accumulated variance of each extracted factor. Brazil, 2013.

Variables	Factor 1	Factor 2	MSA*	Commonality
Coverage of the Family Health Strategy	**0,910**	0,030	0,824	0,829
Family Health Support Center—Modality I	**0,787**	0,299	0,877	0,709
Family Health Support Center—Modality II	**0,775**	-0,189	0,833	0,637
Unemployment rate	0,187	**0,780**	0,694	0,643
Population with an income below ¼ the minimum wage	0,583	**0,793**	0,881	0,969
Gini Index	0,282	**0,896**	0,845	0,883
Municipal Human Development Index	**-0,775**	-0,558	0,868	0,912
The average per capita income	**-0,811**	-0,437	0,848	0,848
Income ratio 20% richer/20% poorer	0,086	**0,898**	0,700	0,813
Income ratio 20% richer/40% poorer	0,131	**0,954**	0,707	0,927
Income ratio 10% richer/40% poorer	0,161	**0,959**	0,680	0,946
Illiteracy rate	**0,839**	0,447	0,892	0,904
Population with less than 4 years of schooling	**0,901**	0,336	0,849	0,925
Population with more than 8 years of schooling	**-0,899**	-0,232	0,726	0,862
Latent root	6,134	5,674		
Variance explained by the factor	43,816	40,527		
Total variance explained	43,816	84,344		

*MSA = Measure of sample adequacy; Extraction Method—Principal Component Analysis; Varimax orthogonal rotation with Kaiser normalization.

For the sensitivity analysis, a factor analysis was performed, as previously described, with 25% of the cases belonging to the database that were selected by simple random sampling.

The sensitivity analysis demonstrated the validity of the construct generated, since two factors with the same variables were extracted, with KMO equal to 0.803; Bartlett’s sphericity test showed *p*<0.001 and the sum of the total explained variance was 84.353%. Thus, the indicator produced consisted of two dimensions, represented by the factors generated, which were called primary health care coverage and unfavorable socioeconomic conditions (factor 1) and economic inequality (factor 2). In order to carry out the analyzes, the factor scores of each factor generated were dichotomized into low and high, based on the median value.

The outcome prevalence was calculated in relation to the individual variables and the indicators produced; then, bivariate Poisson regression analysis with robust variance was performed to estimate the prevalence ratio (PR) and the respective 95% confidence interval (95%CI). Variables that presented *p*≤0.20 were included in the multivariate analysis model. The hierarchical model was adopted, and the variables were entered in the multivariate model according to the increasing order of the p value, and the variables with *p*≤0.10 were maintained in the model. For the final model, only variables with *p*≤0.05 were considered.

All analyses were performed using the software Stata version 14.

## Results

In relation to the total data obtained in the PNS, the results indicate that the prevalence of PWD in Brazil was 7.7% (95%CI 7.3%–8.0%), with a prevalence of intellectual disability of 0.8% (95%CI 0.7%–0.8%), 1.4% physical disability (95%CI 1.3%–1.5%), 2.1% hearing impairment (95%CI 1, 9%–2.2%), and visual impairment of 4.5% (95%CI 4.2%–4.8%). The prevalence of use of some rehabilitation services by PWD in Brazil was 9.2% (95%CI 8.2%–10.2%), while the use of intellectual, physical, auditory, and visual rehabilitation services was, respectively, 2.9% (95%CI 2.4%–3.4%), 3.3% (95%CI 2.7%–3.9%), 2.0% (95%CI 1.6%–2.5%), and 2.8% (95%CI 2.2%–3.4%).

Among individuals who have at least one type of disability, it was found that 50.1% were women, 50.0% were white, 73.4% were over 40 years old, 45.3% were married, 79.0% were literate, 73.7% did not have health insurance, 42.1% had mild limitations, and 43.7% belonged to class C according to the Brazil Criteria for Economic Classification. Regarding the contextual variables, it was observed that the majority lived in a place with a high proportion of PWD by Specialized Rehabilitation Centers, with a low score for the primary health care coverage and the unfavorable socioeconomic conditions factor and a low score for the economic inequality factor (Table 3).

**Table 3:** Prevalence of people with disabilities according to the study variables. Brazil, 2013.

Variables	% (95%CI)
**Gender**	
Male	49.9 (48.6–51.2)
Female	50.1 (48.8–51.4)
**Age**	
0–17 years	7.0 (6.4–7.7)
18–29 years	9.8 (8.9–10.7)
30–39 years	9.8 (9.0–10.7)
40–59 years	34.5 (33.0–36.1)
60 years and over	38.9 (37.3–40.5)
**Race**	
Non-White	50.0 (48.1–51.8)
White	50.0 (48.2–51.9)
**Marital status**	
Married	45.3 (43.6–47.0)
Widowed	12.2 (11.4–13.2)
Separated	6.9 (6.2–7.8)
Single	35.6 (34.1–37.1)
**Literacy**	
Yes	79.0 (77.5–80.3)
No	21.0 (19.7–22.5)
**Socioeconomic level**	
A and B	24.3 (22.4–26.2)
C	43.7 (41.8–45.7)
D and E	32.0 (30.3–33.8)
**Health Insurance**	
No	73.7 (71.8–75.5)
Yes	26.3 (24.5–28.2)
**Limitation**	
Without limitation	34.2 (32.4–36.0))
Mild limitation	42.1 (40.2–43.9)
Severe limitation	23.8 (22.4–25.2)
**Ratio of PWD and the Specialized Rehabilitation Centers—Type II**	
High	68.7 (66.6–70.6)
Low	31.3 (29.4–33.4)
**Ratio of PWD and the Specialized Rehabilitation Centers—Type III**	
High	87.8 (86.7–88.7)
Low	12.2 (11.3–13.3)
**Ratio of PWD and the Specialized Rehabilitation Centers—Type IV**	
High	67.2 (65.2–69.2)
Low	32.8 (30.8–34.8)
**Primary Health Care Coverage and Unfavorable Socioeconomic Conditions**	
Low	61.3 (59.2–63.3)
High	38.7 (36.7–40.8)
**Economic Inequality**	
High	37.9 (35.9–39.8)
Low	62.1 (60.2–64.1)

%: Prevalence; CI95%: 95% confidence interval; PWD: People with disabilities.

Regarding the individual predisposing factors associated with the use of rehabilitation services, it was observed that in the final model, there was a greater use of these services by individuals from 0 to 17 years old (PR = 3.28; 95%CI 2.85–3.78; *p*<0.001), from 18 to 29 years (PR = 2.03; 95%CI 1.73–2.39; *p*<0.001), and from 30 to 39 years (PR = 1.26; 95%CI 1.04–1.53; *p* = 0.018) compared to individuals aged 60 years or older. There was also a greater use among illiterate individuals (PR = 1.24; 95%CI 1.09–1.40; *p*<0.001), socioeconomic level A or B (PR = 1.60; 95%CI 1.35–1.88; *p*<0.001), and socioeconomic status C (PR = 1.34; 95%CI 1.17–1.52; *p*<0.001) compared to individuals of lower socioeconomic status belonging to classes D and E. As for the individual facilitating factors, having health insurance increased the use of rehabilitation services (PR = 1.31; 95%CI 1.15–1.49; *p*<0.001). Regarding the need factors, there was a greater use among individuals with mild limitation (PR = 1.79; 95%CI 1.53–2.08; *p*<0.001) and severe limitation (PR = 3.09; 95%CI 2.64–3.62; *p*<0.001) in relation to individuals without limitation (Table 4).

**Table 4:** Association between access to rehabilitation services for people with disabilities and independent variables. Brazil, 2013.

Brazil						
Variables	Access to rehabilitation services		Bivariate Analysis		Adjusted Model (n = 13.500)	
	%	95%CI	*p*	PR (95%CI)	*p*	PR (95%CI)
**Gender**						
Male	8.9	7.7–10.2		1		
Female	9.4	8.2–10.8	0.693	1.02 (0.92–1.12)		
**Age**						
0–17 years	27.0	23.0–31.3	<0.001	3.53 (3.09–4.04)	<0.001	3.28 (2.85–3.78)
18–29 years	11.8	9.5–14.7	<0.001	1.93 (1.64–2.27)	<0.001	2.03 (1.73–2.39)
30–39 years	9.1	6.9–12.0	0.049	1.21 (1.00–1.48)	0.018	1.26 (1.04–1.53)
40–59 years	7.0	5.6–8.8	0.479	0.94 (0.81–1.09)	0.602	1.04 (0.89–1.20)
60 years and over	7.2	5.9–8.8		1		1
**Race**						
Non-White	9.5	8.2–10.9		1		
White	8.9	7.6–10.4	0.847	1.01 (0.91–1.11)		
**Marital status**						
Married	7.2	5.9–8.8		1		
Widowed	7.4	5.5–9.9	0.559	1.06 (0.86–1.30)		
Separated	8.8	5.3–14.2	0.177	1.18 (0.92–1.50)		
Single	10.1	8.8–11.6	<0.001	1.67 (1.48–1.90)		
**Literacy**						
Yes	8.2	7.2–9.3		1		1
No	11.4	9.6–13.6	<0.001	1.41 (1.26–1.58)	0.001	1.24 (1.09–1.40)
**Socioeconomic level**						
A and B	11.2	9.2–13.4	<0.001	1.38 (1.20–1.58)	<0.001	1.60 (1.35–1.88)
C	8.6	7.5–9.9	<0.001	1.26 (1.12–1.42)	<0.001	1.34 (1.17–1.52)
D and E	8.4	6.7–10.4		1		1
**Health Insurance**						
No	8.4	7.4–9.6		1		1
Yes	11.2	9.3–13.4	<0.001	1.28 (1.14–1.43)	<0.001	1.31 (1.15–1.49)
**Limitation**						
Without limitation	4.6	3.6–6.0		1		1
Mild limitation	9.0	7.7–10.5	<0.001	1.66 (1.44–1.92)	<0.001	1.79 (1.53–2.08)
Severe limitation	16.0	13.6–18–7	<0.001	2.95 (2.56–3.40)	<0.001	3.09 (2.64–3.62)
**Ratio of PWD and the Specialized Rehabilitation Centers—Type II**						
High	9.4	8.0–11.1		1		1
Low	10.0	8.2–12.0	<0.001	1.22 (1.10–1.35)	0.001	1.20 (1.08–1.33)
**Ratio of PWD and the Specialized Rehabilitation Centers—Type III**						
High	9.5	8.0–11.3		1		
Low	11.4	9.0–14.2	0.827	1.01 (0.90–1.13)		
**Ratio of PWD and the Specialized Rehabilitation Centers—Type IV**						
High	9.7	7.6–12.3		1		1
Low	10.0	8.4–11.9	<0.001	1.32 (1.18–1.47)	<0.001	1.29 (1.15–1.44)
**Primary Health Care Coverage and Unfavorable Socioeconomic Conditions**						
Low	8.7	7.5–10.0		1		1
High	9.9	8.4–11.7	<0.001	1.24 (1.12–1.38)	0.011	1.15 (1.03–1.28)
**Economic Inequality**						
High	9.5	8.0–11.3		1		
Low	8.9	7.8–10.2	0.298	1.05 (0.95–1.16)		

CI95%: 95% confidence interval; PWD: People with disabilities.

Deviance Goodness of fit = 5401.49; *p* = 1.000.

As for contextual factors, there was a greater use of rehabilitation services by individuals residing in states with a low proportion of PWD by CER—type II (PR = 1.20; 95%CI 1.08–1.33; *p*<0.001), and CER—type IV (PR = 1.29; 95%CI 1.15–1.44; *p*<0.001) and high scores on the primary health care coverage and unfavorable socioeconomic conditions factor (PR = 1.15; 95%CI 1.03–1.28; *p*<0.001) (Table 4).

## Discussion

This study was conducted based on national data and demonstrated that individual factors related to greater use of rehabilitation services by PWD were being young, not being literate, having a higher socioeconomic level, having health insurance, and having severe limitations. Among the contextual factors, the greatest use was related to living in a place with a low proportion of PWD using rehabilitation services, considering the CERs type II and IV, and with high scores in the primary health care coverage and unfavorable socioeconomic conditions factor. The highest magnitudes of association were observed for age and disability limitation.

Among the individual demographic predisposing factors, there was no association between sex and the outcome. On the other hand, age proved to be an important effect for the use of rehabilitation services. PWD aged 0–17 years used these services three times more than the elderly, the effect being maintained after adjustment for the other variables.

Despite the higher prevalence of PWD observed among people over 40 years of age, paradoxically, there was a dose-response effect of the age group regarding the use of rehabilitation services where the highest prevalence of use of rehabilitation services was observed among the youngest. These results are like those observed by Bernabe-Ortiz et al. [20] in a study conducted in Peru.

The study conducted by Dias et al. [21] sought to understand the perception of PWD in the state of Minas Gerais, in southeastern Brazil, about the quality of rehabilitation services to which they had access and identified that 54.5% of users were under 17 years old.

It is believed that the higher prevalence of access to rehabilitation services for this portion of the population with disabilities may be associated with the fact that they are minors under the care of their parents or guardians. The fact of having a person who accompanies them or takes them to the services could, therefore, justify greater access to these services. Dias et al. [21] observed that among the PWD companions who attended rehabilitation services in Minas Gerais, 74.6% were parents or guardians.

The type and severity of the disability can influence access to services, and in cases of congenital disabilities, multiprofessional follow-up is started as early as possible, which could also justify greater access for people up to 17 years of age, although this has not been the object of analysis in the present research.

There are few studies that discuss PWD access from the perspective of use and are even more limited when it comes to the population under 18 years old. Bright and Kuper [12] conducted a systematic review of the literature on access to health services by PWD in low- and middle-income countries and identified that 20% of the analyzed articles referred to people under 18 years of age and 20% did not differentiate between the age of the research participants, indicating, therefore, the scarcity of research in this area and the need to develop studies that consider this population.

Among the individual social predisposing factors, the influence of socioeconomic level and literacy was observed regarding the greater use of rehabilitation services by PWD. For this population, the effect of skin color on PWD rehabilitation services was not observed, although this is an association found in other studies that assess the use of health services in the general population [22–25].

It was observed in the present study that the socioeconomic level influences the use of rehabilitation services by PWD in which belonging to higher social classes was related to the greater use of these services. These findings are supported by the results found in a study conducted in Peru that sought to assess access to health services by PWD and identified that the PWD of a higher socioeconomic level had 2.21 times more access compared to people of the lowest level [20].

The socioeconomic level is a recognized factor associated with the use of health services observed in several studies conducted for this purpose [25–28].

Health insurance is a facilitating factor for the use of health services [1], is directly related to socioeconomic level [29], is an important indicator of access, and in this study, the effect on the greater use of rehabilitation services, considering that the coverage of health insurance implies reducing financial barriers to the use of health services [30].

The results demonstrate the social inequalities regarding the access to rehabilitation services by PWD, indicating that people with lower income and who are not assisted by health insurance have less access.

Although important changes have been implemented to minimize the effects of social and health inequalities on the living and health conditions of PWD, this population remains on the margins of the labor market, income generation mechanisms, education, and access to social opportunities [31].

The level of education is another important indicator for assessing access to health services, which have a direct and positive correlation with each other [28,32,33]; however, in this research, it was identified that access to rehabilitation services showed an inverse relationship with education.

The World Report on Disability [1] points out that PWDs have lower levels of education compared to people without disabilities. In this sense, it is important to consider that in the context of PWD, the level of education can be related to the degree of severity and limitation imposed by the disability. In a study that sought to characterize visually impaired people seen at a specialized institution, they identified that among blind people, 58.8% had more than 8 years of education, while among people with low vision, this rate was 75.0%, demonstrating the relationship between severity of disability and the level of education [34].

To assess the need for the use of rehabilitation services by PWD, the variable degree of disability limitation was used, and it was observed that the need is configured as a factor associated with greater use. People with disabilities with severe limitations used rehabilitation services 3.1 times more than those without limitations. It is important to note that the inclusion of a variable that assesses the need for use has not mitigated the effect of other factors.

The effect of marital status on the use of rehabilitation services was observed in the bivariate analysis; however, this effect was not maintained after adjusting for the other variables. This finding may have been mitigated by the effect of age in the adjusted analysis, considering that younger individuals, especially those aged 0 to 17 years, were those who had the most access. This effect may also have been mitigated by the severity of the limitation caused by the disability, since more severe limitations can influence such relationships.

In addition to knowledge about individual issues related to PWD access to health services, it is essential to discuss access from different perspectives and to consider contextual aspects.

As for contextual factors, the effect of the greater organization of local health services was observed as a factor influencing the use of rehabilitation services by PWD. The greater availability of public rehabilitation services, represented by the low proportion of PWD by CER types II and IV, was associated with a greater use of rehabilitation services. However, there was no such effect on the proportion of PWD by type III CER.

Specialized rehabilitation centers (CER) are specialized outpatient care points belonging to the care network for people with disabilities (RCPCD) that perform diagnosis, treatment, concession, adaptation, and maintenance of assistive technology. Its organization provides care for people according to the type of disability (physical, intellectual, hearing, and visual) and must combine at least two types of care. The amount of services offered is characterized by its type (II, III, or IV) [2,6,8]. The greater availability of such health equipment contributes directly to greater access by PWD to rehabilitation services.

The CER as components of the RCPCD are articulated with the other points of care under the logic of the organization of health care networks in which primary health care (PHC) is configured as the ordering and care coordinator [7].

It is necessary to highlight that the information referring to CER is related to the year 2014, and this situation may have contributed to the failure to identify the effect on the proportion of PWD per CER type III, considering that the short period since the creation of RCPCD, in 2012, may have had an impact on the reduced number of establishments of this type.

In view of the various variables that relate the influence of the context to the health-disease-care process, especially of the PWD, we opted for the development of composite indicators that would enable such an explanation.

The construction of indicators facilitates the process of understanding, evaluation, and monitoring of determining phenomena and can be applied in relation to the dynamics of the development of populations, spaces, and environments. In this context, composite indicators are presented as tools that reflect multidimensional characteristics because they summarize complex information and compile various information into an indicator, thus contributing to a better understanding of what is under analysis and providing information for policy formulation and for decision-making [35,36].

According to the Organization for Economic Cooperation and Development (OECD) [37], the development of composite indicators and their correct interpretation requires an extremely structured theoretical basis capable of providing elements for the selection of simple indicators that will be combined for consolidation composite indicators that make sense and fit the purpose of the study.

In the process of building indicators, the definition of the variables that will compose the composite indicator constitutes a fundamental step, and it is imperative to avoid arbitrary procedures and use mathematical models or computational techniques. The use of multivariate data analysis techniques such as factor analysis stands out in this process because it summarizes the set of variables and preserves, within limits, the proportion of variation in the original set [35].

For this study, the selection of contextual variables for the construction of the indicators took place under the logic of Andersen’s behavioral model, and the indicators created were the products of the main component analyses. From the variables included in the model, the indicator of primary health care coverage and unfavorable socioeconomic conditions and the indicator of economic inequality were produced.

It was found that high scores for the primary health care coverage and unfavorable socioeconomic conditions demonstrated an effect on the use of rehabilitation services by PWD.

In Brazil, the FHS was the model adopted to strengthen PHC in the country and has been shown to reduce social and health inequities that have been observed for a long time and, therefore, translates into a strategy for the effectiveness of the equity principle since there is greatest coverage of the FHS in the areas with greatest social vulnerability [38]. In this sense, the inverse association between the FHS coverage rates and the level of education, the average per capita income, and the MHDI identified in the composition of the composite indicator is understandable and has already been observed in other studies [39].

From this point of view, PHC as a coordinator and organizer of care under the logic of organizing health care networks plays an important role in the care of PWD both for monitoring carried out by health teams in their territories and for the referral process to rehabilitation services [40].

It was observed that the socioeconomic conditions of the PwD directly reflect on access to rehabilitation services, and that the indicator “economic inequities”, which synthesizes the variables that describe the contextual socioeconomic conditions, was not associated with the outcome. This finding reveals that access to rehabilitation services by PwD suffers a direct impact from individual socioeconomic conditions and is not influenced by contextual conditions.

It is important to point out that the data presented here come from the National Health Survey and allow a wide representation of the Brazilian population since, due to the sampling process used, greater geographic spreading was possible, which positively reflected in the accuracy of the estimates made and which can contribute to the process of formulating public health policies [41,42].

For the development of the PNS, the concept of self-reported disability was used, which can be considered a limitation of the study, considering that the person who participated in the interview could be the participant himself/herself or a proxy and, therefore, the existing subjectivity in relation to the perception of disability and its limitations according to who the respondent was must be taken into account [43].

The research instrument used in the PNS does not allow the evaluation of the different perspectives in relation to access to rehabilitation services by PWD because it considered only the concept of access from the logic of use without considering the factors inherent to this evaluation, such as availability, accessibility, convenience, purchasing power, acceptability and permanence in the service, which is also a limitation.

The evaluation of indicators of access to health services through population surveys is an important strategy for the evaluation of health systems, with repercussions on the process of improving public policies. However, there is a need to qualify this information with the incorporation of data on the quality of care offered. In this sense, it is necessary that in future studies, the dimensions related to the performance of the health system should be considered, such as adequacy, continuity, acceptability, effectiveness, efficiency, security, and respect for the rights of people with disabilities [44].

Finally, when analyzing access to rehabilitation services for people with disabilities in Brazil from the perspective of use and based on data from a population survey, it was observed that in relation to individual factors, access was greater among people who are young, illiterate, of higher socioeconomic level, have health insurance, and have severe limitations, and in relation to contextual factors, access was associated with the fact that they live in places with greater health services and with unfavorable socioeconomic conditions.

The results clarify the social inequities regarding access to rehabilitation services for people with disabilities and show that comprehensive, universal, and equitable care will only be consolidated as soon as the urgency and the need for the formulation and implementation of public policies are recognized, in the different segments, that guarantee the realization of the rights of these people and that face the determinants of health inequalities, which also affect people with disabilities, and which constitute an additional challenge in achieving equity in health for this population.
